# Effect and Safety of Apatinib as Neoadjuvant Therapy in Locally Advanced Differentiated Thyroid Cancer: A Phase 2 Trial

**DOI:** 10.1210/jendso/bvae132

**Published:** 2024-07-12

**Authors:** Kai Qian, Yunjun Wang, Ning An, Chunhao Liu, Kai Guo, Lingyi Yang, Jun Wang, Xiaoyi Li, Zhuoying Wang

**Affiliations:** Department of Head and Neck Surgery, Renji Hospital, School of Medicine, Shanghai Jiaotong University, Shanghai 200001, China; Department of Head and Neck Surgery, Fudan University Shanghai Cancer Center, Shanghai 200032, China; Department of Oncology, Shanghai Medical College, Fudan University, Shanghai 200032, China; Department of Head and Neck Surgery, Gansu Provincial Cancer Hospital, Lanzhou 730050, China; Department of General Surgery, Peking Union Medical College Hospital, Chinese Academy of Medical Sciences & Peking Union Medical College, Beijing 100730, China; Department of Head and Neck Surgery, Renji Hospital, School of Medicine, Shanghai Jiaotong University, Shanghai 200001, China; Department of Head and Neck Surgery, Renji Hospital, School of Medicine, Shanghai Jiaotong University, Shanghai 200001, China; Department of Head and Neck Surgery, Gansu Provincial Cancer Hospital, Lanzhou 730050, China; Department of General Surgery, Peking Union Medical College Hospital, Chinese Academy of Medical Sciences & Peking Union Medical College, Beijing 100730, China; Department of Head and Neck Surgery, Renji Hospital, School of Medicine, Shanghai Jiaotong University, Shanghai 200001, China

**Keywords:** local advanced differentiated thyroid cancer, neoadjuvant therapy, apatinib, phase II trial, multicenter study

## Abstract

**Context:**

Presently, there is a paucity of prospective clinical trials investigating neoadjuvant therapy for locally advanced thyroid cancer.

**Objective:**

This study was a multicenter, open-label, single-arm, phase II trial evaluating the efficacy and safety of apatinib as neoadjuvant therapy in patients with local advanced differentiated thyroid cancer (DTC).

**Methods:**

Patients were treated with preoperative apatinib over a course of 2 to 4 cycles, culminating in surgical resection. The primary endpoints were objective response rate (ORR) and disease control rate (DCR); the secondary endpoints were the rate of R0 surgery, alterations in serum thyroglobulin levels, disease-free survival, and adverse events (AEs).

**Results:**

A total of 14 patients who met the inclusion criteria were administered neoadjuvant apatinib. Among these, 13 patients underwent surgical procedures following apatinib treatment and were enrolled in the ITT population. The ORR was 53.8% and the DCR was 100%. Of the patients, 84.6% received R0 surgery, while the remaining 15.4% underwent R1 resection. Predominant among the observed AEs were hypertension, hand–foot syndrome, hepatic dysfunction, proteinuria, and hypothyroidism, with no instances of grade 4 or 5 AEs reported. Subsequent to surgery, patients were followed up for a median period of 34 months, during which disease progression occurred in 5 individuals (35.7%), encompassing 3 cases of locoregional recurrences and 2 cases of distant metastases.

**Conclusion:**

Apatinib may be an effective agent in the use of neoadjuvant therapy for locally advanced DTC. Patients may therefore benefit from surgical outcomes and their long-term prognosis.

Over the preceding decades, there has been a global surge in the incidence of thyroid cancer [1]. In 2020, the worldwide tally of new thyroid cancer cases exceeded 586 000, ranking it 11th among all malignancies [2]. Among these cases, differentiated thyroid cancer (DTC) has a generally favorable prognosis and its postsurgical clinical remission can be achieved in over 90% of all thyroid cancer instances. Consequently, the overall mortality rate associated with thyroid cancer remains relatively low [2]. Nonetheless, a subset of patients presents with locally advanced disease upon their initial clinical assessment, introducing complexities in treatment strategies, particularly when tumor lesions infiltrate vital adjacent structures. This clinical scenario is far from rare. Existing literature reports that approximately 13% to 15% of DTC cases exhibit primary lesions invading critical structures [3, 4]. The involvement of these crucial structures necessitates invasive surgical procedures, augmenting the risk of residual disease, recurrence, and, in extreme cases, forfeited surgical options. On one hand, nonradical tumor resection stands as a significant determinant of unfavorable prognosis in patients with DTC [5]. On the other hand, extensive surgical excision can precipitate a precipitous decline in the patient's quality of life. Therefore, the pursuit of safe and radical surgical interventions for patients with locally advanced thyroid cancer assumes paramount significance.

Previous studies have attempted to improve the resection rates in cases of locally advanced DTC through the implementation of neoadjuvant chemotherapy [6, 7]. However, the widespread recommendation of this approach has been hindered by the lack of large-sample, high-quality data supporting its efficacy. Presently, kinase inhibitors, representing a vanguard in the domain of targeted medications, have progressively assumed a prominent role in the management of advanced DTC. Nevertheless, it is noteworthy that while targeted drugs and their associated clinical trials have found utility in the adjuvant treatment of advanced thyroid carcinomas [8], their application in the context of neoadjuvant therapy remains relatively nascent. Reports concerning neoadjuvant therapy predominantly comprise case reports and case series [9, 10], with prospective clinical investigations in this domain remaining relatively rare at present [11]. The potential role of targeted drugs as neoadjuvant therapies thus beckons further exploration.

Apatinib stands as a highly selective inhibitor, specifically targeting vascular endothelial growth factor receptor 2 [12]. Preclinical investigations have unveiled its potent antitumor activity against thyroid cancer cells [13, 14]. In the REALITY study, the potential of adjuvant apatinib was explored within the cohort of patients afflicted with radioactive iodine–refractory (RAIR)-DTC [15]. The objective response rate (ORR) was 54.3% and the disease control rate (DCR) was 95.7% in the apatinib group, significantly better than the placebo group.

This study was a prospective phase II trial designed to assess both the effectiveness and safety of apatinib when utilized as a neoadjuvant therapy in patients with locally advanced DTC.

## Materials and Methods

### Study Design

This investigation was designed as a multicenter, open-label, single-arm, phase II trial, specifically focusing on the administration of neoadjuvant apatinib to patients with locally advanced DTC, carried out across 4 hospitals in 3 cities (Shanghai, Beijing, and Lanzhou) in China. The study was conducted in accordance with Good Clinical Practice guidelines and the Declaration of Helsinki and was reviewed and approved by the institutional ethics committee of Renji Hospital affiliated to Shanghai Jiaotong University School of Medicine (KY2019-178), Fudan University Shanghai Cancer Center (1709176-15), Peking Union Medical College Hospital (B271), and Gansu Provincial cancer hospital (A201901090001). The study was registered in Chinese Clinical Trial Registry (https://www.chictr.org.cn) (ChiCTR OIC 17013425). All participating patients provided and signed the informed consent as a testament to their voluntary participation and understanding of the study's objectives and procedures.

### Patients

Key eligibility criteria encompassed the following: age ≥18 years old, ≤75 years old; newly diagnosed histologically or cytologically confirmed DTCs that have not been previously treated with surgery or other treatments; local advanced DTC defined as American Joint Committee on Cancer 8th edition T3b or T4 staging with significant invasion of at least 1 perineural structure including larynx, trachea, pharynx, esophagus, carotid artery, and prevertebral fascia, which were difficult to achieve R0/1 resection based on computed tomography scan assessment; at least 1 measurable lesion in accordance with the Response Evaluation Criteria in Solid Tumors (RECIST) version 1.1; Eastern Cooperative Oncology Group performance status ranging from 0 to 2; meeting contraceptive and pregnancy related requirements; patients voluntarily entered the study and signed informed consent form. A panel of 4 high-volume surgeons who perform more than 500 thyroid cancer surgeries per year from the Department of Head and Neck Surgery involved in the study determined eligibility for patients with locally advanced thyroid cancer. Key exclusion criteria encompassed the following: individuals with tumors completely surrounding the carotid arteries; other histological subtypes of thyroid cancer; participation in other drug clinical trials; extensive distant metastases; allergy to the drug featured in this protocol; uncontrolled severe infections or other severe uncontrolled diseases; long-term unhealed wounds; uncontrolled hypertension, grade III/IV coronary heart disease, arrhythmia, or heart failure; abnormal coagulation function or bleeding tendency; history of other malignancies within the past 5 years, except cured skin basal cell carcinoma or cervical carcinoma in situ.

The sample size of this study was calculated using Simon's 2-stage design with a type I error rate α (1-sided) of 0.05 and a power of 80%. Compared with the ORR of 30% in previous studies, the ORR of this study was expected to be 65%. Under this assumption, 7 patients would be enrolled in the first stage, and if at least 2 patients achieved complete response (CR) or partial response (PR), the second stage would be continued. In the second stage, 7 patients were enrolled. The treatment regimen would be considered successful if 7 or more responses of all the 14 patients were observed.

### Procedures

The study was divided into 2 distinct phases: neoadjuvant therapy followed by surgery. During the neoadjuvant phase, patients were scheduled to undergo 2 to 4 cycles (4 weeks as a cycle) of oral apatinib at a dosage of 500 mg daily. Evaluations of treatment efficacy were conducted following the second, third, and fourth treatment cycles. On the premise of excluding surgical contraindications, patients for whom the panel of 4 high-volume surgeons evaluated and deemed the timing appropriate were subsequently slated for surgical intervention. The timing of surgery after neoadjuvant therapy was considered in this study to be partial response or tumor regression when R0/1 resection was expected to achieved. Patients who were considered not to be suitable for surgery continued to be under observation or subjected to ongoing treatment. Following surgery, apatinib treatment was discontinued. Subsequent therapeutic avenues were contingent upon the specifics of the surgical procedure and the postoperative pathology. For patients with disease progression, unacceptable toxicity, or refusal to continue treatment during neoadjuvant therapy, alternative options including surgery were made available, following which patients transitioned into the survival follow-up phase.

Computed tomography scans were performed to assess treatment response and radiologic tumor response was assessed according to RECIST measurement criteria (version 1.1). The adverse events (AEs) were evaluated according to National Cancer Institute's CTCAE v4.0.

### Outcomes

The primary endpoint of the study was ORR and DCR. The secondary endpoints included the rate of achieving R0 surgery, alterations in serum thyroglobulin (Tg) levels, long-term treatment response, disease-free survival (DFS), and safety.

### Statistical Analyses

ORR was calculated as the percentage of patients who achieved CR or PR as a result of at least 1 evaluation before disease progression. DCR was defined as the proportion of CR and PR and stable disease among all the patients included. The evaluation of long-term treatment response was stratified into 4 distinct categories in accordance with the 2015 ATA guideline [16]: excellent response (ER), biochemical incomplete response, structural incomplete response (SIR), and indeterminate response. Efficacy analyses were carried out within the intention to treat (ITT) population and safety analyses were based on all patients who received at least 1 cycle of apatinib.

Rate of DFS was calculated using the Kaplan–Meier method and the prognostic factors were analyzed using the log-rank test. All tests were 2-tailed. *P* values less than .05 were considered to be statistically significant. All the data obtained from the study were analyzed qualitatively and quantitatively by using SPSS 25.0 (IBM Corp, Armonk, NY).

## Results

### Patient Characteristics

**Figure 1.**
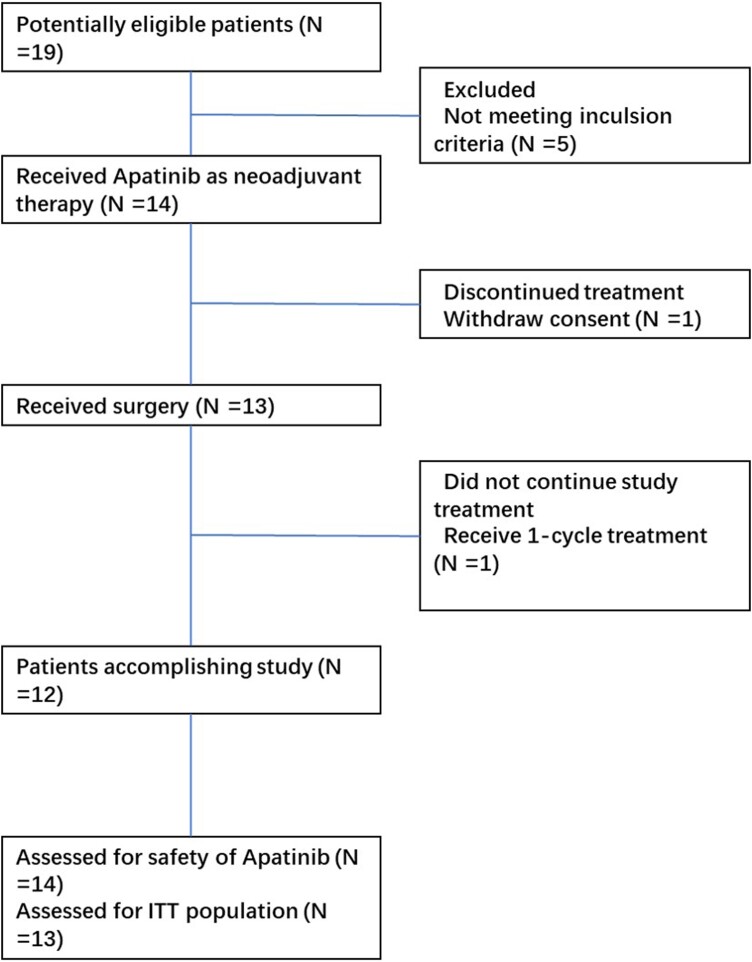
Consolidated Standards of Reporting Trials (CONSORT) flow diagram.

Between November 2017 and July 2021, a total of 14 patients underwent neoadjuvant apatinib treatment. The essential information and clinical characteristics of the patients are shown in Table 1. The majority of participants in this study were female (71.4%), with an average age of 50.9 years old. All enrolled patients had received a pathological confirmation of lateral cervical lymph node metastasis before surgery. Additionally, 1 patient, suspected of harboring a small solitary lung metastatic lesion based on imaging, was also included in the study. One patient withdrew consent and did not proceed to surgery. Among the remaining participants, 1 patient underwent surgery after a single cycle of apatinib treatment. Eleven patients completed 2 cycles of apatinib, while 1 patient completed 3 cycles before undergoing surgical resection. Thirteen patients were enrolled as the ITT population (Fig. 1).

**Table 1.** Baseline characteristics of patients who received neoadjuvant apatinib

Characteristics	No. of patients (%)
Sex	
Female	10 (71.4)
Male	4 (28.6)
Age, average (range), y	50.9 (28-70)
ECOG performance status	
0	13 (92.9)
1	1 (7.1)
Pathology	
Classic	10 (71.4)
Infiltrative follicular	2 (14.3)
Clear cell	1 (7.1)
Unknown	1 (7.1)
cT stage	
T3b	1 (7.1)
T4a	11 (78.6)
T4b	2 (14.3)
cN stage	
N1b	14 (100)
cM stage	
M0	13 (92.9)
M1	1 (7.1)
AJCC 8th cTNM stage	
I	9 (64.3)
II	1 (7.1)
III	3 (21.4)
IVB	1 (7.1)

Abbreviations: AJCC, American Joint Committee on Cancer; ECOG, Eastern Cooperative Oncology Group.

### Efficacy Outcomes

**Figure 2.**
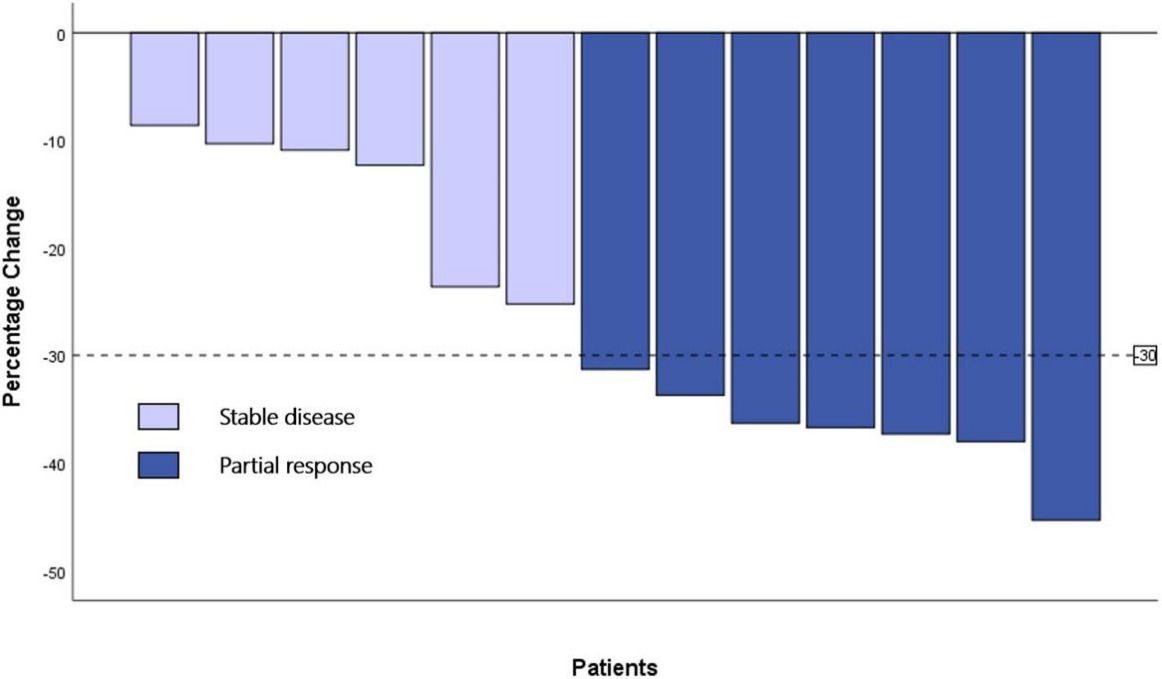
Waterfall plot for best percentage change from baseline in the sum of the longest diameters of target lesions after neoadjuvant apatinib treatment.

**Figure 3.**
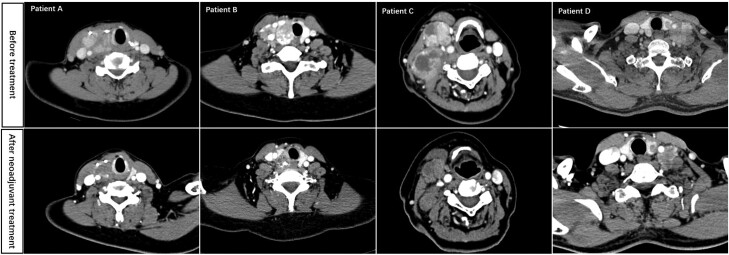
The computed tomography scans showing tumor changes before and after neoadjuvant treatment. Patient A: Thyroid tumor decreased in size by 37.3%. Patient B: The target lesions decreased 36.3%. The primary lesion developed liquefaction necrosis. The right internal jugular vein was no longer compressed by lymph nodes after 2 cycles of apatinib treatment. Patient C: The lymph node at the right neck decreased in size by 33.7%. Patient D: The metastatic lymph node at the left neck decreased and the left common carotid artery was no longer surrounded by lymph nodes.

Within the ITT population, none of the cases achieved a complete response and 7 patients exhibited a partial response with an overall response rate of 53.8% (95% CI 25.1-80.8). The remaining 6 patients displayed stable disease and the DCR was 100%. Response rates for each patient are shown in Fig. 2. Typical cases are shown in Fig. 3.

As for secondary endpoint, the rate of R0 surgery was 84.6% (95% CI 54.6-98.1) and the other 2 patients underwent R1 resection. Tumor invasion was common in these patients. Recurrent laryngeal nerve involvement was identified in 10 patients and 4 of them had the recurrent laryngeal nerve severed as a result. Other frequently involved organs included the strap muscles (6/13), trachea (4/13), esophagus (3/13), and jugular vein (3/13), with 1 patient requiring tracheotomy.

**Figure 4.**
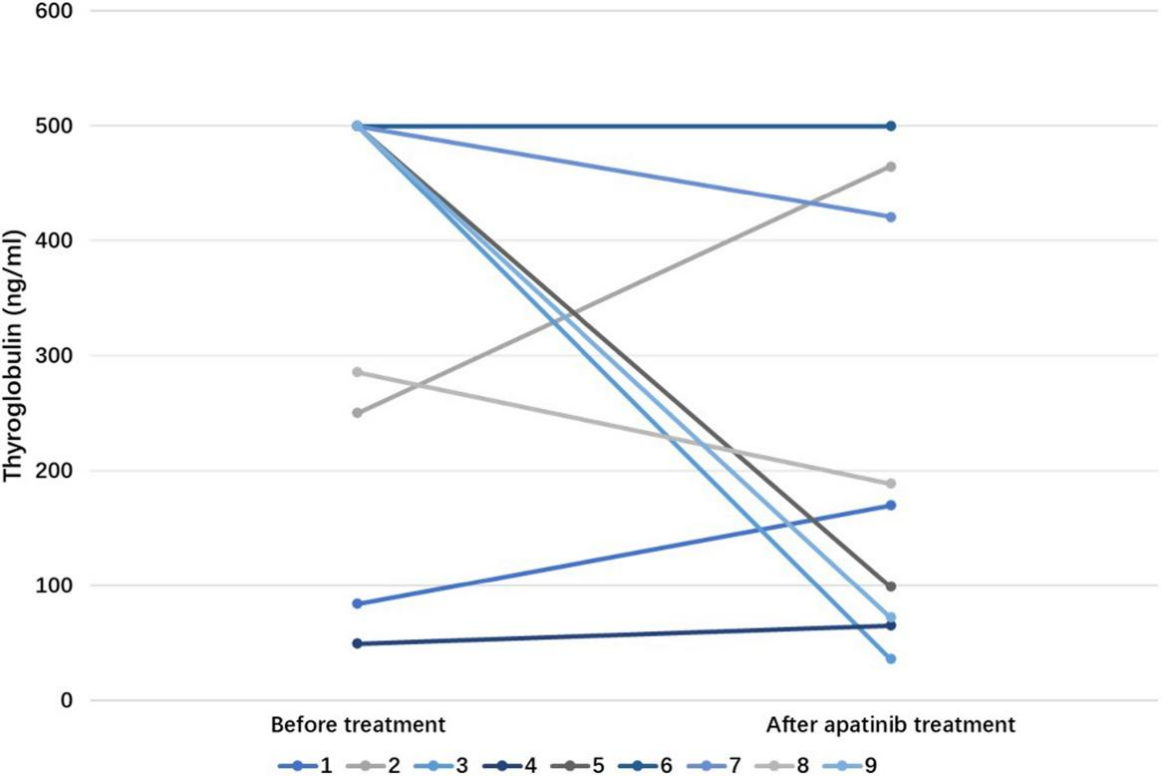
Alterations in serum Tg levels before and after apatinib therapy in patients with normal antithyroglobulin antibodies.

Furthermore, 4 patients exhibited anti-Tg antibodies outside the normal range. Among the 9 patients for whom Tg levels could be assessed, 5 experienced a decline in Tg levels following neoadjuvant therapy (Fig. 4).

### Safety and Feasibility

In the safety analysis encompassing all 14 patients, every participant encountered at least 1 AE. In total, 52 AEs were reported (Table 2). There were no grade 4 or 5 AEs, and 10 (19.2%) AEs attained the grade 3 severity level. Among the most frequently encountered AEs were hypertension, hand–foot syndrome, and hepatic dysfunction. Hypertension and hand–foot syndrome were predominant among the grade 3 AEs. Six individuals (42.9%) necessitated either a dose reduction or treatment interruption due to treatment-related AEs of any grade, mainly including 2 (14.3%) with hand–foot syndrome, 2 (14.3%) with thrombocytopenia, 1 (7.1%) with hemoptysis, and 1 (7.1%) with proteinuria. One patient (7.1%) received only 1 cycle of apatinib and discontinued apatinib owing to diarrhea and hypertension, then accepted surgery.

**Table 2.** Adverse events reported during apatinib therapy

Adverse events	No. of patients	
	Grade 1/2 N %	Grade 3 N %
Hypertension	7 (50.0)	3 (21.4)
Hand–foot syndrome	5 (35.7)	2 (14.3)
Hepatic dysfunction	6 (42.9)	1 (7.1)
Hypothyroidism	5 (35.7)	0 (0)
Proteinuria	4 (28.6)	1 (7.1)
Thrombocytopenia	3 (21.4)	1 (7.1)
Diarrhea	2 (14.3)	1 (7.1)
Abdominal pain	2 (14.3)	0 (0)
Hemoptysis	1 (7.1)	1 (7.1)
Pharyngalgia	1 (7.1)	0 (0)
Anemia	1 (7.1)	0 (0)
Hematochezia	1 (7.1)	0 (0)
Leukopenia	1 (7.1)	0 (0)
Hematuria	1 (7.1)	0 (0)
Vomiting	1 (7.1)	0 (0)
Debilitation	1 (7.1)	0 (0)

### Follow-up and Survival Analyses

**Figure 5.**
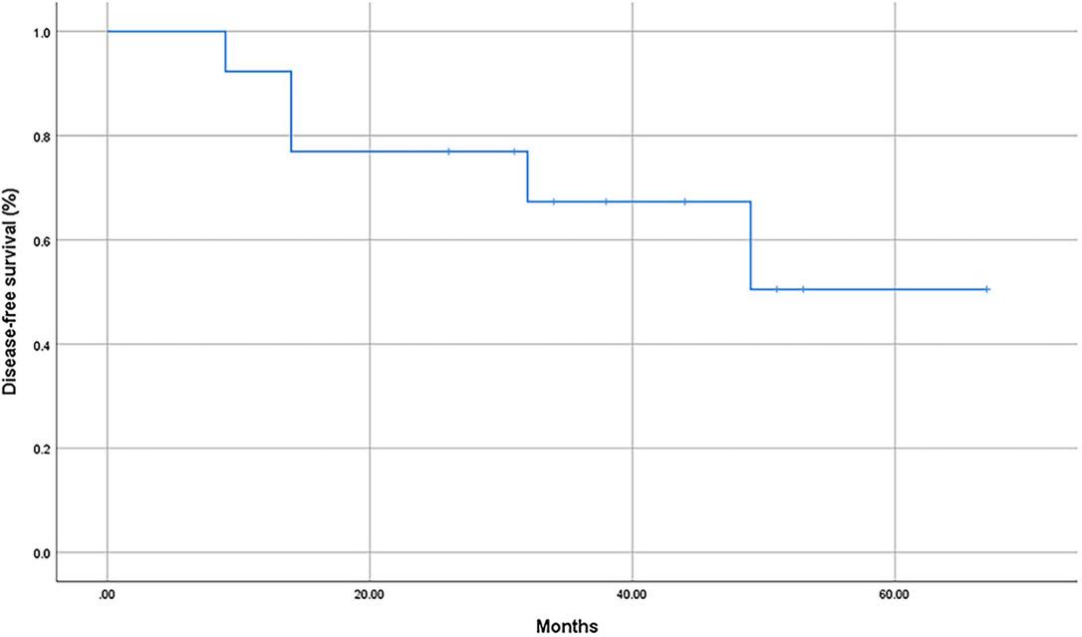
Kaplan–Meier curve of disease-free survival of all the patients enrolled in the ITT population.

The entire cohort of 13 patients was followed up for 9 to 67 months, with a median follow-up time of 34 months. After surgery, 12 patients received iodine-131 treatment. Disease progression occurred in 5 (35.7%) during the follow-up period, including 3 cases of locoregional recurrences and 2 cases of distant metastases (Table 3). These 5 patients had SIR. Among the remaining patients, 3 (23.1%) achieved ER, 4 (30.8%) exhibited biochemical incomplete response, and 1 (7.7%) had indeterminate response. No fatalities were recorded at the culmination of the final follow-up period. The Kaplan–Meier curve of DFS is shown in Fig. 5 and median DFS was not reached.

**Table 3.** Demographic and treatment characteristics of 14 cases

Case	Sex	Age at presentation	Stage at diagnosis	Response to apatinib	Cycles	Surgical response	Iodine-131 treatment	Duration of follow-up (months)	Recurrence/metastasis
1	Female	48	T4aN1bM0	PR	2	R0 resection	Yes	67	No
2	Female	53	T4bN1bM0	PR	2	R1 resection	Yes	32	Lymph node
3	Female	70	T4aN1bM0	PR	2	R0 resection	Yes	9	Lung
4	Male	56	T4aN1bM0	PR	2	R0 resection	Yes	14	Lymph node
5	Male	28	T4aN1bM0	PR	2	R1 resection	Yes	53	No
6	Female	62	T4aN1bM1	SD	2	R0 resection	Yes	49	Lung
7	Male	53	T4aN1bM0	SD	2	R0 resection	Yes	14	Lymph node
8	Female	44	T4aN1bM0	SD	2	R0 resection	Yes	51	No
9	Female	53	T4aN1bM0	SD	2	R0 resection	Yes	44	No
10	Female	34	T4aN1bM0	SD	1	R0 resection	Yes	34	No
11	Female	32	T3bN1bM0	SD	2	R0 resection	Yes	26	No
12	Male	62	T4bN1bM0	PR	2	R0 resection	Yes	38	No
13	Female	50	T4aN1bM0	PR	3	R0 resection	No	31	No
14	Female	68	T4aN1bM0	—	—	—	—	—	—

Abbreviations: PR, partial response; SD, stable disease.

## Discussion

In recent years, neoadjuvant therapy has emerged as an important approach in facilitating radical surgical interventions for patients with locally advanced tumors, including thyroid cancer. The fundamental objective of neoadjuvant therapy is to diminish the tumor stage, increase the possibility of radical resection, and enhance the overall quality of life for patients through preoperative treatment [17, 18]. Though endeavors to employ neoadjuvant chemotherapy [6] and neoadjuvant radiotherapy [19, 20] for patients bearing locally advanced lesions have been underway since the 1990s, these efforts faced constraints imposed by drug limitations and technological challenges, thus failing to gain widespread attention at the time. Presently, with the development of targeted therapy and immunotherapy, neoadjuvant therapy for thyroid cancer has made some breakthroughs. A number of case reports or case series for neoadjuvant purposes have been published in different pathological types of thyroid cancer, such as BRAF V600E–mutated anaplastic thyroid carcinoma [21-23] and medullary thyroid cancer with RET mutation [24, 25].

However, there remains a conspicuous scarcity of prospective clinical trials concerning neoadjuvant therapy for thyroid cancer [11]. To the best of our knowledge, we report the first multicenter, prospective phase II clinical trial of neoadjuvant therapy in initially treated local advanced DTC. Notably, in addition to safety and short-term effects, our study also focused on long-term prognosis. In this trial, preoperative treatment comprised 500 mg of oral apatinib daily for 2 to 4 cycles before surgery, referring to the treatment of advanced gastric adenocarcinoma [26] and RAIR-DTC [15]. These 2 clinical trials have showed that a high dose (850 mg or 750 mg) of apatinib is more likely to bring AEs with a high incidence and dose suspension. Therefore, a dose of 500 mg was adopted in this study. Regarding the assessment of efficacy, we found most patients achieved optimal results following 2 cycles of treatment, with only 1 patient undergoing a third cycle of apatinib. In the REALITY study, the median time to objective response of apatinib in RAIR-DTC was 1.9 months. In previous studies of neoadjuvant therapy, the duration of targeted drugs was about 2 to 6 months [11, 27, 28]. These data were also similar to our study.

To date, the application of neoadjuvant therapy is still in its infancy. Within our study, the DCR was 100% after neoadjuvant therapy, with 53.8% of patients exhibiting a partial response. The DCR and ORR of this study mirror those reported in the REALITY study [15]. These findings provide initial insight into the potential merits of neoadjuvant therapy. By diminishing the tumor burden, this approach enhances the prospects of executing radical and safe surgical interventions. Every patient within the ITT population achieved an R0/1 resection, underscoring the role of neoadjuvant apatinib in improving the feasibility of surgery and contributing to a good rate of radical resection.

Among the cohort of 14 patients administered apatinib, a total of 52 AEs were reported. The majority of these documented AEs manifested as grade 1 or 2, characterized by mild symptoms amenable to resolution through symptomatic interventions. The most common grade 3 AEs were hypertension and hand–foot syndrome, which are consistent with those unveiled in the REALITY study [15]. These AEs exhibited a propensity for mitigation following a reduction in drug dosage and the application of symptomatic interventions, often achieving resolution within a brief temporal span, typically around 1 week. In addition, no surgical complications directly attributable to apatinib were identified during the course of this investigation.

With a median follow-up of 34 months, these patients achieved a relatively good prognosis, with the absence of any fatalities and a limited cohort of just 5 individuals displaying structural incomplete responses. Besides, 5 patients did not attain the status of ER, but their Tg levels remained stable throughout the follow-up duration. Existing literature underscores the poor prognosis could associated with locally advanced lesions [29, 30], emphasizing the pivotal role of complete tumor excision in prognostic improvement [31]. In a retrospective study, locally advanced DTC patients who underwent R2 resection exhibited a 5-year disease-specific survival rate of 67.9% [32]. In survival analysis, we did not find any independent factors related to DFS, which may be attributed to the small sample size. However, we found that in 75% (3/4) patients with SIR and normal anti-Tg antibodies, the Tg level increased after neoadjuvant treatment with apatinib. This might be a potential predictor of long-term outcomes in such patients.

A standardized criterion for administering neoadjuvant therapy in cases of locally advanced thyroid cancer has yet to be established. Presently, the patient profiles that might derive maximal benefits from neoadjuvant therapy remain unclear, primarily due to the lack of large-scale clinical trials. On the other hand, neoadjuvant therapy entails inherent risks and uncertainties. For instance, the potential for tumor progression during the preoperative phase might inadvertently delay the surgical intervention; the side effects associated with neoadjuvant therapy can render certain patients unsuitable candidates for surgery and so on. Further complexities arise concerning the optimal selection of therapeutic agents and the precise timing of surgical procedures, even in instances where neoadjuvant therapy demonstrates efficacy in tumor reduction. Clarification of these aspects remains a pressing challenge.

Several limitations of our study should be taken into account. Firstly, the small sample size hampers the feasibility of subgroup analysis and constrains the efficacy of survival analysis. Secondly, our study design was single arm, lacking a control group, which consequently limits direct comparisons of safety data. Thirdly, the mean follow-up duration, at 34 months, is relatively brief, precluding long-term insights. Lastly, genetic testing was not incorporated into the study protocol, thus the genetic characteristics were not presented.

### Conclusions

Apatinib as neoadjuvant therapy for locally advanced DTC was feasible and majority of patients achieved R0/1 resection. Patients may therefore benefit from both surgical outcomes and long-term prognosis. The adverse reactions were controllable, and no increased surgical complications were observed. These results suggest that apatinib may be an effective agent in the use of neoadjuvant therapy for locally advanced DTC. Further assessment through large-scale randomized clinical trials is merited to corroborate this treatment strategy.

## Data Availability

Some or all datasets generated during and/or analyzed during the current study are not publicly available but are available from the corresponding author on reasonable request.

## Clinical Trial Information

Clinical Trial Registry number and website: The study was registered in Chinese Clinical Trial Registry (https://www.chictr.org.cn), unique identifier: ChiCTR OIC 17013425 (November 17, 2017).

## Abbreviations

AE: adverse event
CR: complete response
DCR: disease control rate
DFS: disease-free survival
DTC: differentiated thyroid cancer
ER: excellent response
ITT: intention to treat
ORR: objective response rate
PR: partial response
RAIR: radioactive iodine–refractory
SIR: structural incomplete response
Tg: thyroglobulin
